# Wireless optogenetics protects against obesity via stimulation of non-canonical fat thermogenesis

**DOI:** 10.1038/s41467-020-15589-y

**Published:** 2020-04-07

**Authors:** Kazuki Tajima, Kenji Ikeda, Yuji Tanabe, Ella A. Thomson, Takeshi Yoneshiro, Yasuo Oguri, Marc D. Ferro, Ada S. Y. Poon, Shingo Kajimura

**Affiliations:** 1 UCSF Diabetes Center, San Francisco, CA 94143 USA; 2 Eli and Edythe Broad Center of Regeneration Medicine and Stem Cell Research, San Francisco, CA 94143 USA; 3 Department of Cell and Tissue Biology, University of California, San Francisco, CA 94143 USA; 4 Department of Electrical Engineering, Stanford University, Stanford, CA 94305 USA; 5 Department of Materials Science and Engineering, Stanford University, Stanford, CA 94305 USA; 6 Chan Zuckerberg Biohub, San Francisco, CA 94158 USA; 7 Present Address: Department of Molecular Endocrinology and Metabolism, Tokyo Medical and Dental University, 1-5-45, Yushima, Bunkyo-ku, Tokyo, 113-8510 Japan

**Keywords:** Fat metabolism, Molecular medicine

## Abstract

Cold stimuli and the subsequent activation of β-adrenergic receptor (β-AR) potently stimulate adipose tissue thermogenesis and increase whole-body energy expenditure. However, systemic activation of the β3-AR pathway inevitably increases blood pressure, a significant risk factor for cardiovascular disease, and, thus, limits its application for the treatment of obesity. To activate fat thermogenesis under tight spatiotemporal control without external stimuli, here, we report an implantable wireless optogenetic device that bypasses the β-AR pathway and triggers Ca^2+^ cycling selectively in adipocytes. The wireless optogenetics stimulation in the subcutaneous adipose tissue potently activates Ca^2+^ cycling fat thermogenesis and increases whole-body energy expenditure without cold stimuli. Significantly, the light-induced fat thermogenesis was sufficient to protect mice from diet-induced body-weight gain. The present study provides the first proof-of-concept that fat-specific cold mimetics via activating non-canonical thermogenesis protect against obesity.

## Abstract (web summary)

Cardiovascular risks of cold exposure and the subsequent activation of the β3-AR pathway limit the application of beige fat thermogenesis for the treatment of obesity. Here, the authors show that optogenetics light-activated Ca2+ cycling in adipocytes triggers a fat-specific “cold-mimetic” thermogenesis response protecting mice against diet-induced obesity.

## Introduction

Non-shivering thermogenesis by thermogenic fat cells (brown and beige fat) plays a significant role in the regulation of whole-body energy homeostasis. Recent studies have illuminated the therapeutic potential of thermogenic fat for the treatment of metabolic disorders because impaired adipose thermogenesis is tightly associated with the development of obesity and insulin resistance, whereas activation of the thermogenic pathway potently improves metabolic health^1,2^.

The best-known stimulus of adipose tissue thermogenesis is cold: following cold exposure, noradrenaline (NA) released from the sympathetic nerve system (SNS) activates the β3-adrenergic receptor (β3-AR) and its downstream signaling pathway, including PKA and p38MAPK, which triggers adipose tissue thermogenesis through uncoupling protein 1 (UCP1) as well as UCP1-independent mechanisms^3–6^. Accordingly, extensive efforts have been made to develop β3-AR agonists as an anti-obesity medication that stimulates adipose tissue thermogenesis. Consistent with the model, a selective β3-AR agonist, mirabegron, significantly promotes adipose tissue thermogenesis and resting metabolic rate by approximately 200 kcal per day in adult humans^7^. However, a major hurdle for applying β3-AR agonists to the treatment of obesity is that, besides the poor bioavailability, pharmacological β3-AR activation at an effective dose to achieve thermogenesis inevitably increases blood pressure as well^8,9^. Accordingly, identifying alternative pathways that stimulate fat thermogenesis, while avoiding cardiovascular risks, could lead to effective and safe therapeutic interventions for obesity.

The recent identification of non-canonical UCP1-independent thermogenesis may offer unique opportunities to enhance adipose tissue thermogenesis^10,11^. We previously reported a UCP-independent mechanism in beige fat that involves ATP-dependent Ca^2+^ futile cycling through Sarco/endoplasmic reticulum Ca^2+^-ATPase2b (SERCA2b) and Ryanodine Receptor 2 (RyR2)^10^. The Ca^2+^ cycling thermogenesis is evolutionally conserved in humans, mice, and also pigs, a mammalian species that lack a functional UCP1 protein^10^. Notably, enhanced Ca^2+^ cycling by S107, a chemical stabilizer for the RyR2-Calstabin interaction, potently stimulated UCP1-independent thermogenesis and protected *Ucp1* null mice from hypothermia following cold exposure^10^. A limitation in the study, however, was that S107 was systemically administered to mice, such that we were not able to exclude the possibility that other tissues than the adipose tissue, such as skeletal muscle, might contribute to UCP1-independent thermogenesis. Thus, adipocyte-specific manipulation of the Ca^2+^ cycling pathway would critically test the therapeutic potential of non-canonical fat thermogenesis for the treatment of obesity.

In this regard, optogenetics is a powerful tool for temporal and spatial manipulation of neuronal or cellular activities in vivo. The conventional optogenetics studies required fiber optic tethering and/or large head-mounted receivers, such that it limits the application to metabolic studies in general. In contrast, a recent advance in wirelessly powered optogenetics devices enabled an efficient and stable light delivery into peripheral nerves of freely behaving animals^12^. Accordingly, we develop a wireless optogenetics device that is implantable to the subcutaneous adipose tissue of mice. The device is distinct from the previous attempts in that it can stimulate peripheral cells (i.e., adipocytes) rather than neurons. By employing this implantable wireless device, the present study reports that light-activated Ca^2+^ cycling in adipocytes sufficiently stimulates non-canonical thermogenesis and protects mice against diet-induced obesity.

## Results

### Development of a wireless optogenetics device for adipose tissue

**Fig. 1:**
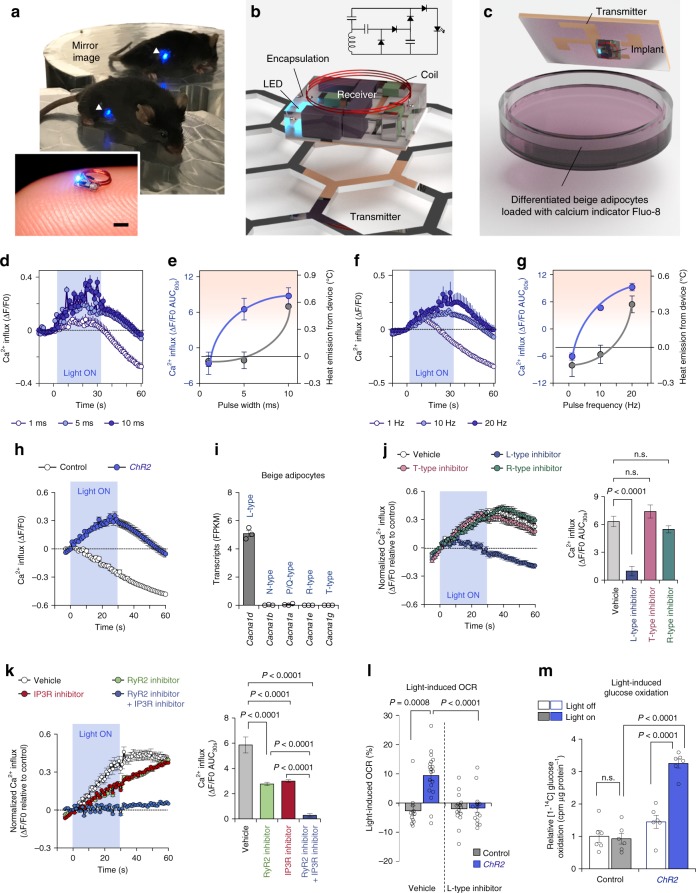
Wireless optogenetics implant stimulates Ca^2+^ influx in adipocytes. **a** A representative image of a freely behaving mouse with the implantable wireless optogenetics device in the inguinal WAT (arrow heads). Scale bar, 1 mm. **b** Three-dimensional illustration of the implantable wireless optogenetics device and the circuit diagram. **c** Schematic of optogenetic studies in cultured cells. **d**–**g** Real-time changes in intracellular Ca^2+^ influx following optogenetic light stimulations with indicated pulse width (**d**) or pulse frequency (**f**). **d** Cells stimulated with pulse width at 1 ms, *n* = 104; at 5 or 10 ms, *n* = 140. **f** Cells stimulated with pulse frequency at 1 or 10 Hz, *n* = 140; at 20 Hz, *n* = 134. Quantification of intracellular Ca^2+^ influx in *ChR2*-expressing adipocytes stimulated with indicated pulse width (**e**) or pulse frequency (**g**) and the heat emission from the device. The data of heat emission from the device were derived from Supplementary Fig. 1c for (**e**) or 1d for (**g**). **h** Real-time changes in intracellular Ca^2+^ influx following optogenetic light stimulation. Vector control, *n* = 123; *ChR2*, *n* = 128. **i** Expression of indicated voltage-gated Ca^2+^ channels in isolated beige adipocytes (E-MTAB-3978). *n* = 3. **j** Real-time changes (left) and quantification (right) in intracellular Ca^2+^ influx following optogenetic light stimulation. *ChR2* with vehicle, *n* = 128; *ChR2* with L-type inhibitor, *n* = 140; *ChR2* with R-type inhibitor, *n* = 155; *ChR2* with T-type inhibitor, *n* = 134; Vector control with vehicle, *n* = 139. **k** Real-time changes (left) and quantification (right) in intracellular Ca^2+^ influx following optogenetic light stimulation. *ChR2* with vehicle, *n* = 105; *ChR2* with RyR2 inhibitor, *n* = 131; *ChR2* with IP_3_R inhibitor, *n* = 120; *ChR2* with RyR2 inhibitor and IP_3_R inhibitor, *n* = 140; Vector control with vehicle, *n* = 140. **l** Quantification of light-stimulated OCR following optogenetics light stimulation. Vector control with vehicle, *n* = 12; Vector control with L-type inhibitor, *n* = 15; *ChR2* with vehicle, *n* = 19, *ChR2* with L-type inhibitor, *n* = 15. **m** Glucose oxidation in differentiated adipocytes expressing *ChR2* or vector control with or without optogenetics light stimulation. *n* = 6. Data were analyzed by one-way ANOVA (**j**–**l**) or two-way ANOVA (**m**) by Tukey’s post hoc test. All Data are expressed as means ± s.e.m. n.s. not significant.

We previously utilized the strong localization of electromagnetic energy at low gigahertz frequencies to develop wireless optogenetics devices with dimensions on the order of a few millimeters^12^. However, there is very limited space between the skin and subcutaneous fat tissue to accommodate the device. Thus, we optimized the height of the device to be sub-millimeter (Fig. 1a, inset), such that they can be inserted bilaterally into the subcutaneous inguinal adipose tissue of mice (Fig. 1a and Supplementary Movie 1). The device is much smaller (2 mm^3^, 20 mg) than our previously reported devices, whereas the efficiency of LED remained the same as the previous study (approximately 19%) and the power transferred to the implant at the output of the rectifier across the surface of the resonant cavity approximately varied from 1.5 to 3 mW (time-averaged cavity input power of 150 mW, 5% duty cycle)^12^. In addition, the efficiency of the rectifier was between 20% and 60% (Supplementary Fig. 1a). This wireless optogenetic device can be implanted entirely underneath the skin, while the light source was not blocked by the antenna or other circuit components. The device consists of a tiny power-harvesting coil (2-mm diameter) with terminals connected to a receiving circuit configured with a rectifier to drive a blue micro-LED to activate channelrhodopsin 2 (ChR2) (Fig. 1b). The attenuation coefficient in adipose tissues was lower than that in the brain (adipose tissues, *μ*′_s_ = 0.8 mm^−1^; Brain, *μ*′_s_ = 1.6 mm^−1^), suggesting that the wireless LED device can stimulate *ChR2*-expressing cells in a broader area of adipose tissues relative to the brain (Supplementary Fig. 1b). A primary bi-layer of acrylic and Parylene-C was used to encapsulate the device to electrically insulate the circuitry and to resist biological degradation for chronic studies (Supplementary Fig. 1c). The implanted devices are powered and controlled using an aluminum resonant cavity with a surface lattice of hexagons^13^. In addition to the in vivo test setup, we also developed an in vitro setup to rapidly depolarize cultured cells that express *ChR2* upon exposure to blue light. The optogenetic device was powered by a crossed-slot antenna^14^, and they were placed on top of a glass-bottom dish (Fig. 1c).

By using these systems, we determined the optimal light pulses that stimulate intracellular Ca^2+^ influx with negligible heat emission from the device. To this end, we performed optogenetic stimulation in differentiated beige adipocytes expressing *ChR2* with fixed 10-Hz frequency and 1, 5, or 10 ms pulse widths (1%, 5%, and 10% duty cycle, respectively) (Fig. 1d). For each set of pulse parameters, we also measured heat emission from the wireless device implanted into the inguinal WAT of wild-type mice (Supplementary Fig. 1d). Based on the measurements in intracellular Ca^2+^ influx and tissue temperature, we found that a pulse width of 5 ms yielded potent intracellular Ca^2+^ influx, whereas the same light pulses did not trigger heat emission from the device in the inguinal WAT of mice (Fig. 1e). Subsequently, we fixed the pulse width to 5 ms and varied the pulse frequency to 1, 10, or 20 Hz (0.5%, 5%, and 10% duty cycle, respectively) in beige adipocytes expressing *ChR2* and the inguinal WAT of wild-type mice (Fig. 1f and Supplementary Fig. 1e). These results showed that pulse width of 5 ms and 10-Hz frequency (5% duty cycle) was optimal to stimulate intracellular Ca^2+^ flux with negligible heat emission from the device in the adipose tissue (Fig. 1g). The light-stimulated intracellular Ca^2+^ influx was observed at a pulse width of 500 μs as long as the duty cycle was maintained at 5% (Supplementary Fig. 1f, g). The light-triggered intracellular Ca^2+^ influx is due to optogenetic activation of ChR2 because the optimized light-pulses with 10-Hz frequency and 5-ms pulse width potently increased intracellular Ca^2+^ influx in beige adipocytes expressing *ChR2*, whereas no change was seen in control cells expressing an empty vector (Fig. 1h and Supplementary Movie 2). Of note, the gradual decline in intracellular Ca^2+^ level was owed to a relatively rapid decay of the calcium indicator Fluo-8, and thus the intercellular Ca^2+^ influx was normalized by the decay rate of each assay in the following experiments.

ChR2 is the light-gated, inwardly rectifying cation channel that transports protons, and both monovalent (Na^+^, K^+^) and divalent cations (Ca^2+^, Mg^2+^)^15^. Thus, we next examined the mechanism by which light-activated membrane depolarization triggers intracellular Ca^2+^ influx in beige adipocytes. It is worth noting that our previous transcriptomics data^16^ found that L-type voltage-dependent Ca^2+^ channel (*Cacna1d*) was highly expressed in isolated beige adipocytes, whereas N, P/Q, R, and T-type voltage-dependent Ca^2+^ channels were nearly absent (Fig. 1i). This observation caught our attention because pharmacological inhibition of L-type and R-type Ca^2+^ channels blunts optogenetics-induced Ca^2+^ flux in cultured beta-cells^17^. Accordingly, we treated beige adipocytes with pharmacological inhibitors of voltage-dependent Ca^2+^ channels and examined optogenetics-induced intracellular Ca^2+^ influx. We found that pharmacological inhibition of L-type voltage-dependent Ca^2+^ channel by the specific inhibitor, isradipine, completely blunted the increase in intracellular Ca^2+^ influx following optogenetic stimulation. On the other hand, blockade of R-type and T-type voltage-dependent Ca^2+^ channels by SNX-482 and NNC55-0396, respectively, did not affect the optogenetics effect (Fig. 1j). These data suggest that the L-type voltage-gated Ca^2+^ channel mediates the light-induced membrane depolarization and triggers Ca^2+^ cycling in beige adipocytes.

Next, we determined the source of the intracellular Ca^2+^ signal in response to optogenetic stimulation of ChR2. Our previous study showed that RyR2 and inositol 1,4,5-trisphosphate receptors (IP_3_R1 and IP_3_R2) are expressed in beige adipocytes, and that activation of RyR2 enhances Ca^2+^ cycling thermogenesis in beige fat^10^. Accordingly, we asked the extent to which inhibition of Ca^2+^ release from the ER inhibits light-induced Ca^2+^ flux. To this end, we treated *ChR2*-expressing beige adipocytes with inhibitors for RyR2 (Ryanodine) and/or IP_3_R (2-APB). Following ChR2 activation by blue light, we found that each inhibitor partly, but significantly, reduced intracellular Ca^2+^ flux. Notably, the combination of the two inhibitors completely abolished the light-induced Ca^2+^ signal (Fig. 1k). These data suggest that Ca^2+^ release from the ER via RyR2 and IP_3_R is required for light-activated Ca^2+^ cycling in beige fat.

We subsequently found that optogenetic activation of Ca^2+^ cycling was sufficient to activate beige fat thermogenesis without any external stimuli, such as noradrenaline (NA): the measurement of cellular oxygen consumption rate (OCR) in *ChR2*-expressing beige adipocytes significantly increased following light stimulation. Consistent with the data that L-type voltage-dependent channel blocker (isradipine) inhibited light-induced Ca^2+^ influx, blockade of L-type channel completely blunted the light-induced OCR (Fig. 1l). Importantly, optogenetic activation of Ca^2+^ cycling was sufficient to stimulate glucose oxidation in *ChR2*-expressing beige adipocytes, but not in control cells (Fig. 1m).

### Optogenetics device stimulates fat thermogenesis via SERCA2

**Fig. 2:**
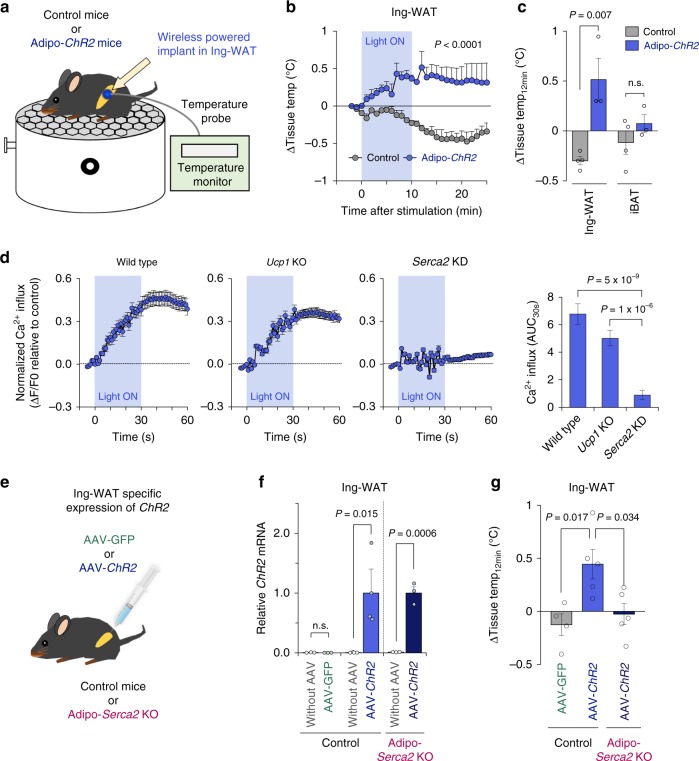
Wireless optogenetics stimulates Ca^2+^ cycling fat thermogenesis through SERCA2. **a** Schematic illustration of the tissue temperature recording in device-implanted mice. **b** Real-time changes in the inguinal WAT temperature of Adipo-*ChR2* mice and littermate controls following optogenetic stimulation. Control, *n* = 4; Adipo-*ChR2*, *n* = 3. **c** Quantification of light-induced thermogenesis in the inguinal WAT and iBAT of Adipo-*ChR2* mice and littermate controls in **b**. Control, *n* = 4; Adipo-*ChR2*, n = 3. **d** Real-time changes in intracellular Ca^2+^ influx in wild-type, *Ucp1* KO, and *Serca2*KD beige adipocytes following optogenetics light stimulation. Wild-type, n = 134 for both groups; *Ucp1* KO control, *n* = 140; *Ucp1* KO-*ChR2*, *n* = 139; *Serca2*KD control, *n* = 139; *Serca2*KD-*ChR2*, *n* = 134. **e** Schematic illustration of the experiment. AAV-GFP or AAV-*ChR2* were injected into the inguinal WAT of Adipo-*Serca2* KO and age-matched control mice. **f** mRNA expression of *ChR2* in the left side (with AAV) and the right side (without AAV) of inguinal WAT. mRNA expression was normalized to *36B4*. Control with AAV-GFP, *n* = 3 for with or without AAV; Control with AAV-*ChR2*, *n* = 4 for with or without AAV; Adipo- *Serca2* KO with AAV-*ChR2*, *n* = 3 for with or without AAV. **g** Quantification of light-stimulated thermogenesis in the inguinal WAT of mice in **f**. Control with AAV-GFP, *n* = 4; Control with AAV-*ChR2*, *n* = 5; Adipo- *Serca2* KO with AAV-*ChR2*, *n* = 5. Data were analyzed by two-way repeated measures ANOVA (**b**), unpaired two-sided *t*-test (**c**, **f**), or two-way ANOVA (**d**) or one-way ANOVA (**g**) by Tukey’s post hoc test. All Data are expressed as means ± s.e.m. n.s. not significant.

We next determined if this system sufficiently enhanced adipose tissue thermogenesis in vivo. To do this, we generated Adipo-*ChR2* mice that expressed ChR2 selectively in mature adipocytes by crossing Adiponectin-Cre mice with Ai32 mice that expressed an improved ChR2 protein (ChR2 H134R) fused to EYFP only in Cre-expressing cells (Supplementary Fig. 2a). Following implantation of the wireless optogenetics devices into the inguinal WAT of Adipo-*ChR2* or littermate control mice, we monitored changes in the adipose tissue temperature using the micro-temperature probes (Fig. 2a). During the temperature recording, we needed to keep the temperature probe within the inguinal adipose tissue. As such, the inguinal adipose tissue lost some insulation (i.e., the fur and skin), resulting in a modest reduction in adipose tissue temperatures of control mice. In contrast, optogenetic activation of ChR2 potently increased the inguinal WAT temperature of Adipo-*ChR2* mice (Fig. 2b). Notably, the light-stimulated adipose tissue thermogenesis occurred selectively in the inguinal WAT and lasted for approximately 70 min even after the end of light stimulation, although the duration would include both thermogenesis and heat retention (Fig. 2c and Supplementary Fig. 2b).

Our previous study shows that Ca^2+^ cycling thermogenesis is an ATP-dependent process in beige fat, and is near completely absent in interscapular BAT (iBAT)^10^. We reasoned that brown adipocytes express low levels of the ATP synthase and thus possess low ATP synthesis capacity^5,18^. Hence, we examined the tissue specificity of light-activated thermogenesis in iBAT. To this end, we implanted the wireless optogenetic device into the interscapular region of Adipo-*ChR2* mice and control mice. Subsequently, we monitored temperature changes in the iBAT in response to light activation of ChR2 (Supplementary Fig. 2c). We found that optogenetic activation of ChR2 in the interscapular region did not trigger thermogenesis in the iBAT (Supplementary Fig. 2d), suggesting that light-activated thermogenesis is selective to the subcutaneous WAT.

Next, we examined if optogenetic activation of ChR2 triggers intracellular Ca^2+^ influx in *Ucp1* null adipocytes because Ca^2+^ cycling in beige adipocytes occurs independently of UCP1^10^. We found that the optimized light-pulses potently increased intracellular Ca^2+^ influx in *Ucp1* null beige adipocytes to a similar degree to wild-type cells expressing *ChR2* (Fig. 2d and Supplementary Fig. 2e, f). In contrast, the optogenetic stimulation with the same frequency and width completely failed to alter intracellular Ca^2+^ influx in SERCA2-depleted adipocytes (Fig. 2d). Consistent with the results, NA stimulated intracellular Ca^2+^ influx in wild-type and *Ucp1* null beige adipocytes, whereas NA failed to do so in SERCA2-depleted adipocytes (Supplementary Fig. 3a). This increase in Ca^2+^ flux was accompanied by increased OCR in wild-type and *Ucp1* null beige adipocytes but not in SERCA2-depleted adipocytes (Supplementary Fig. 3b). Together, these data suggest that optogenetic activation of ChR2 triggers Ca^2+^ cycling through SERCA2 in a UCP1-independent manner.

As an alternative approach to stimulate light-activated fat thermogenesis, we delivered the adeno-associated virus (AAV) expressing *ChR2* or GFP control into the inguinal WAT. To further determine the requirement of Ca^2+^ cycling for light-activated thermogenesis, we expressed ChR2 in Adipocyte-specific *Serca2* KO mice that we generated in our previous study^10^ (Fig. 2e, f). Consistent with the results in Adipo-*ChR2* mice, optogenetic activation of ChR2 potently stimulated thermogenesis in the inguinal WAT expressing AAV-mediated ChR2. However, the light-activated thermogenesis was completely abrogated in the inguinal WAT of Adipo-*Serca2* KO mice that expressed ChR2 (Fig. 2g). No change was detected in the skeletal muscle temperature of all the mice (Supplementary Fig. 3c). Together, these results suggest that optogenetic activation of ChR2 triggers adipose tissue thermogenesis in vivo, and that this effect requires Ca^2+^ cycling through SERCA2 and RyR2/IP_3_R.

### Optogenetics device increases whole-body energy expenditure

**Fig. 3:**
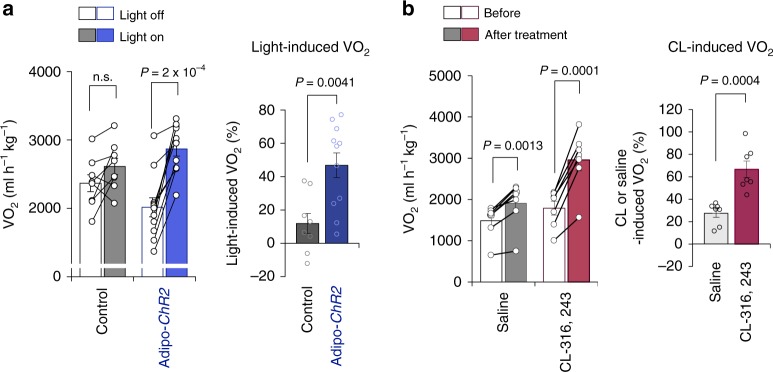
Wireless optogenetics increases whole-body energy expenditure at thermoneutrality. **a** Whole-body oxygen consumption rate (VO_2_) of Adipo-*ChR2* mice and littermate controls following optogenetics light stimulation at thermoneutrality (left). The quantification of light-stimulated VO_2_ is shown on the right graph. Control, *n* = 8; Adipo-*ChR2*, *n* = 11. **b** Whole-body VO_2_ following a single administration of β3 adrenergic receptor agonist (CL-316,243 at 0.01 mg kg^−1^) or vehicle (saline) at thermoneutrality. The quantification of CL-316,243 or saline-induced VO_2_ of wild-type mice is shown on the right. saline, *n* = 7; CL-316,243, *n* = 7. Data were analyzed by two-way repeated-measures ANOVA followed by post-hoc paired/unpaired *t*-test with Bonferroni’s correction (left in **a**–**b**) or unpaired two-sided *t*-test (right in **a**–**b**). All Data are expressed as means ± s.e.m. n.s. not significant.

Next, we asked if optogenetic activation of beige fat thermogenesis could lead to an increase in whole-body energy expenditure without external cold stimuli. To this end, we implanted the wireless optogenetic device into control and Adipo-*ChR2* mice under a thermoneutral condition at 30 °C, where the SNS-mediated regulation of thermogenesis was minimized. Subsequently, we measured whole-body oxygen consumption rate (VO_2_) of control and Adipo-*ChR2* mice during 30 min before and after a 10-min optogenetic stimulation. We found that optogenetic stimulation of fat thermogenesis significantly increased VO_2_ of Adipo-*ChR2* mice by 34.9% relative to control mice (Fig. 3a). During the metabolic cage studies, we did not detect any difference in the locomotor activity between control and Adipo-*ChR2* mice (Supplementary Fig. 3d). These results suggest that the increase in whole-body energy expenditure stems from enhanced thermogenesis in the inguinal WAT. Notably, this increase in whole-body energy expenditure by a 10-min optogenetic stimulation was equivalent to the effect seen after a single administration of β3-AR agonist (CL-316,243) at the dose of 0.01 mg kg^−1^ in mice under the same thermoneutral condition: a single administration of CL-316,243 increased VO_2_ by 39.3% relative to mice received a saline injection at 30 °C (Fig. 3b).

### Light-activated thermogenesis protects against diet-induced obesity

**Fig. 4:**
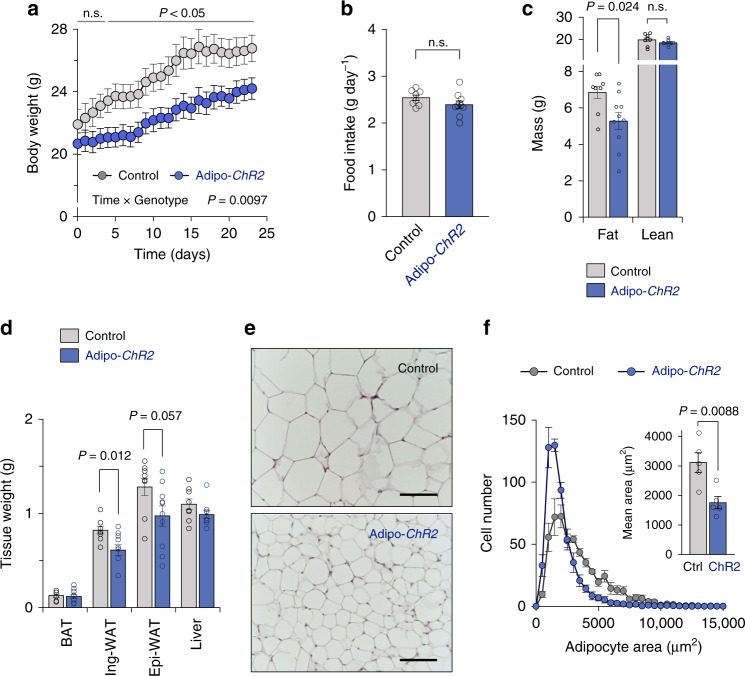
Light-induced adipose tissue thermogenesis prevents diet-induced obesity. **a** Changes in body weight of Adipo-*ChR2* mice and littermate controls. Control, *n* = 8; Adipo-*ChR2*, *n* = 10. **b** Food intake of Adipo-*ChR2* mice and littermate control mice in **a**. Control, *n* = 8; Adipo-*ChR2* *n* = 10. **c** Body composition of Adipo-*ChR2* mice and littermate controls in **a**. Control, *n* = 8; Adipo-*ChR2*, *n* = 10. **d** Tissue-weight of adipose tissues and liver of Adipo-*ChR2* mice and littermate controls in **a**. Control, *n* = 8; Adipo-*ChR2*, *n* = 10. **e** Representative images of hematoxylin and eosin (H&E) staining of the inguinal WAT from Adipo-*ChR2* mice (lower panel) and the littermate controls (upper panel). Scale bars,100 μm. **f** Histogram (left panel) and quantification (right panel) of adipocyte size in the inguinal WAT in **e**. Control, *n* = 5; Adipo-*ChR2*, *n* = 5. Data were analyzed by two-way repeated-measures ANOVA followed by post hoc unpaired *t*-test with Bonferroni’s correction (**a**), unpaired two-sided *t*-test (**b**–**d**, and **f**). All data are expressed as means ± s.e.m. n.s. not significant.

The above results motivated us to test the hypothesis that optogenetic stimulation of adipose tissue thermogenesis protects against diet-induced obesity. Our data suggest that the light-induced Ca^2+^ cycling thermogenesis requires SERCA2; however, the expression of SERCA2 in the adipose tissue decreases under an obese state^10^. Thus, control and Adipo-*ChR2* mice received β3-agonist treatment for 5 days prior to high-fat diet studies to induce SERCA2 expression in the inguinal WAT (Supplementary Fig. 4a). Subsequently, wireless optogenetic devices were implanted into the inguinal WAT of these mice. Five days following the device implantation, both control and Adipo-*ChR2* groups received 10 min light pulses with 10-Hz frequency and 5-ms pulse width once a day for 23 days on a high-fat diet (60% HFD). We found that Adipo-*ChR2* mice gained significantly less body-weight than control mice at 4 days and thereafter following light-activated thermogenesis on a HFD (*P* = 0.0097 by two-way ANOVA followed by unpaired two-sided *t*-test). At the end of the HFD experiments, Adipo-*ChR2* mice gained significantly less body-weight than control mice by 2.6 g on average (9.6% body-weight loss relative to controls) (Fig. 4a). During the study, no significant difference was detected in the food intake between the two groups (Fig. 4b).

The light-induced changes in body-weight were due to reduced fat mass in Adipo-*ChR2* mice, but not to changes in lean mass (Fig. 4c). Specifically, the inguinal WAT of Adipo-*ChR2* mice was significantly smaller than that of control mice at the end of HFD studies (Fig. 4d). A similar trend was seen in the epididymal WAT mass, while no change was noted in the iBAT and liver weight. Histological analyses found that the inguinal WAT of Adipo-*ChR2* mice contained significantly smaller adipocytes than control mice (Fig. 4e, f). Since beige fat biogenesis is associated with reduced adipose tissue inflammation and fibrosis formation^19–21^, we examined if light-activation of thermogenesis in the inguinal WAT altered the cellular composition of adipocytes as well as inflammatory and fibrotic cells. However, transcriptional analysis of the inguinal WAT of control and Adipo-*ChR2* mice found no significant difference in the expression of genes involving in adipose tissue inflammation, fibrosis, and beige fat biogenesis (Supplementary Fig. 4b–d). Of note, device implantation did not alter the expression of pro-inflammatory genes in the inguinal WAT relative to sham-operated mice (Supplementary Fig. 4e). Because our metabolic study was limited to 4 weeks of HFD, mice did not develop notable glucose intolerance at the end of the HFD study. Similarly, hepatic triglyceride levels remained within a normal range. Thus, we did not find a significant difference in systemic glucose tolerance and hepatic triglyceride contents between control mice and Adipo-*ChR2* mice at the end of HFD studies (Supplementary Fig. 4f, g).

## Discussion

Emerging evidence suggests the importance of beige fat in the regulation of energy homeostasis, while its relative contribution to whole-body energy metabolism has been a topic of debate^5,22^. This is mainly because accurate estimation of beige fat’s contribution relative to other tissues, such as skeletal muscle and BAT, is challenging when animals are exposed to external stimuli (e.g., cold) or hormonal cues (e.g., NA). In this regard, the wireless optogenetic technology enabled us to selectively stimulate ChR2-expressing adipocytes in the subcutaneous WAT of mice. Notably, our data suggest that a 10-min stimulation of Ca^2+^ cycling thermogenesis per day, i.e., fat-specific “cold-mimetics”, potently increased total energy expenditure of adult mice by ~35% under a thermoneutral condition. This effect was substantial and was equivalent to the effect of a single administration of β3-AR agonist, CL-316,243, at a dose of 0.01 mg kg^−1^ on total energy expenditure at thermoneutrality. Given the difference in body size/composition and pharmacokinetics between mice and humans, the above estimation cannot be translated directly to adult humans; however, a recent study in humans demonstrates that oral administration of mirabegron, a FDA-approved β3-AR selective agonist, at 200 mg increased resting metabolic rate by ~200 kcal^7^. According to a weight-loss prediction model in humans, every 100 kJ (23.9 kcal) per day increment of energy expenditure would lead to an eventual 1 kg body weight change with half of the predicted weight change in one year (i.e., ~4 kg for 200 kcal day^−1^ increase) and 95% of the change in 3 years^23^.

Our data suggest that light-activation of ChR2 triggers Ca^2+^ release from the ER via RyR2 and IP_3_R, which leads to Ca^2+^ cycling and increased ATP consumption by SERCA2. Mechanistically, previous studies in SERCA1 (the muscle form of SERCA) reported that thermogenesis occurs when Ca^2+^ transport is uncoupled from ATP hydrolysis by SERCA1 under certain conditions, such as low ADP/ATP ratio or low Ca^2+^ binding affinity of SERCA (i.e., uncoupling of ATP hydrolysis and Ca^2+^ transport). When a Ca^2+^ gradient is formed across the ER or SR membrane, heat generation by the uncoupled SERCA1’s ATPase activity can be up to 24.7 kcal/ATP mol^24,25^. Mechanistically, it has been demonstrated that micropeptides, such as Phospholamban, Sarcolipin, DWORF, Myoregulin, control the Ca^2+^ affinity of SERCA in cardiac muscle and skeletal muscle^26–30^; among them, only sarcolipin has been shown to have the SERCA uncoupling activity. However, our RNA-seq data in beige adipocytes of mice and humans did not detect any of these micropeptides are expressed in beige adipocytes^31^. Thus, we aim to search for as-of-yet unknown regulators of SERCA2b ATP-hydrolysis activity in beige adipocytes.

It is notable that optogenetic activation of ChR2 triggers a sustained increase in Ca^2+^ cycling thermogenesis in beige fat. It has been known that activation of L-type channel or changes in ER Ca^2+^ concentration triggers Ca^2+^ release via RyR2 and IP3R^32–34^. Also, we found that RyR2 overexpression significantly increased UCP1-independent thermogenesis following NA treatment, whereas blockade of RyR2 and IP3R attenuates light-evoked intracellular Ca^2+^ flux and UCP1-independent thermogenesis^10^. Hence, it is conceivable that enhanced ER Ca^2+^ release via RyR2/IP3R significantly contributes to the sustained Ca^2+^ cycling thermogenesis through several mechanisms. For instance, PKA signaling, a major downstream signal of β3-AR, is known to phosphorylate RyR2, leading to enhanced Ca^2+^ release^35^. In addition, the cluster size of RyR2 controls Ca^2+^ release in smooth muscle cells^36^. Thus, future studies need to determine the mechanisms through which light- or NA-stimulation alters the open state of RyR2 and/or IP3R in adipocytes, such as time-dependent changes in the phosphorylation and the cluster size.

In contrast to previous efforts to manipulate neural activities, the present study stimulated adipocytes by the wireless optogenetic device. A current technical limitation, however, is that the device is stable in the adipose tissue for up to 4 weeks in vivo. An improved optogenetic device with a longer period of high-fat diet study would warrant future studies to test the effect of light-activated adipose tissue thermogenesis on systemic glucose homeostasis, insulin sensitivity, and hepatic steatosis. To this end, better encapsulation techniques should be applied to extend the longevity of the device. Besides, we may further smooth out the PCB corners and submerge the implant into epoxy in order to have a homogeneous thickness of insulation around the implants. Future designs on the device will also allow for temperature sensing and power control to minimize the heat emission from the device, such that light pulses of higher duty cycles can be applied to enhance Ca^2+^ cycling thermogenesis.

## Methods

### Wireless optogenetics device

The wireless optogenetics device consists of two main parts. The first part is a 2-turn power receiving coil (2-mm diameter) made of copper wire (200-μm diameter). It extracts RF energy coupled from the cavity to the mouse in the in vivo experiments. The second part is the rectifier and micro-LED. The rectifier converts the RF energy into direct current to drive the micro-LED. It was implemented as a two-stage voltage doubling circuit using Schottky diodes (Skyworks, SMS7630-061). The micro-LED (Cree, DA2432) is in bare die form to save space. All components were bonded to a circuit board made of Rogers Printed Circuit Board (PCB) material for ease of cutting. The dimension of the circuit board is 1.6 by 1.8 mm. The overall height of the implant is dominated by the height of the capacitors, which are 0.3 mm. The entire device was encapsulated in epoxy and coated by Parylene-C to form a biocompatible and impermeable membrane protecting the internal electronics. Parylene-C was deposited using an SCS Labcoater 2 to a thickness of 2 µm to ensure pinhole-free films. A 3-(trimethoxysilyl) propyl methacrylate (A-174 Silane) solution was used as an adhesion promoter to maximize the adhesion of the film to the surface of the device. To estimate the accelerated rate test of the wireless LED device, we simulated degradation of the implants using reactive accelerated aging (RAA) techniques^37^. Briefly, the implants were soaked into a solution of 20 mM hydrogen peroxide H_2_O_2_ in Phosphate Buffer Saline (PBS) and were heated up to 60 °C, which mimics an immune system attack with accelerated reactive oxygen species (ROS) chemical reaction^38^. We refreshed the solution with additional H_2_O_2_ every 11 h for 5 days in order to maintain proper chemical reactions. At the end of the experiment, the implants were inspected under a microscope. To determine the light attenuation in adipose tissue, we measured the transmission of 470 nm light from the CREE micro-LED in freshly isolated adipose tissues (inguinal and epididymal WAT) and the brain of mice using a Thorlabs PM100D optical power meter. Tissues were placed between two glass slides, and the thickness was measured using a caliper. Transmission percentages were calculated as a ratio of the optical power measured through 2 mm of tissue or an air gap, to the power measured directly through the glass slides with no air gap. Based on the measurement of the attenuation coefficient in adipose tissues and a previous report showing that the threshold of stimulation for ChR2 is approximately 1 mW mm^−2^^39^, it is estimated that the wireless LED device can stimulate *ChR2*-expressing cells in a surrounding region with a radius greater than 0.75 mm, a broader area of adipose tissues relative to the brain.

### Optogenetics device for cultured cells

A board with 24 LED’s was fabricated to enable optogenetic testing with a seahorse cell culture plate. Blue LED’s (BIVAR R20BLU-4-0045) were used, which have a peak wavelength of 470 nm, and a typical luminous intensity of 8000 mcd. The LED’s were soldered on a prototyping board, in series with a 47-Ohm resistor to limit current. One LED was aligned with the center of each well on the seahorse plate. 4-Volt supplies were used to power the LED’s. An Arduino program was used to control the LED’s, with a frequency of 10 Hz and a duty cycle of 50%. 2N7000 MOSFET’s were used as switches to selectively control the power to the LED’s.

### Animals

All animal experimental procedures were performed in compliance with all relevant ethical regulations applied to the use of small rodents. The animal protocol was approved by the UCSF Institutional Animal Care and Use Committee. For the generation of adipocyte-specific express channelrhodopsin-2 (Adipo-*ChR2*) mice, Ai32 (RCL-ChR2 (H134R) /EYFP) mice (male, Bl6 background) were obtained from the Jackson Laboratory (Stock No: 024109) and crossed to *Adiponectin*-Cre mice. We also used male adipocyte-specific *Serca2* KO mice (Adipo-*Serca2* KO, *Adiponection*-Cre × *Atp2a2*^flox/flox^) in the Bl6 background as described previously^10^. For metabolic cage studies, we obtained male Bl6 mice from the Jackson Laboratory (Stock No: 000664). All the mice had free access to food and water, 12 h light–dark cycles, and were caged at an ambient temperature of 22 °C or thermoneutrality (30 °C). For the study of stimulation with optogenetic wireless implant on a high-fat diet, Adipo-*ChR2* mice (8–9 weeks old, male) and the littermate control mice (male) were treated with β3 adrenergic receptor agonist (CL-316, 243 (Sigma)) at 1 mg kg^−1^ for 5 days prior to the implantation surgery to induce SERCA2 expression in the adipose tissue. The mice were implanted with optogenetic wireless devices on the bilateral inguinal adipose tissues under isoflurane anesthesia (2–3%). Five days after the implantation, all the mice received light-pulses with 10-Hz frequency and 5-ms pulse width for 10 min once a day on a high-fat diet (D12492, Research diet). In this protocol, we minimize handling stress, and no change in animal behavior and food intake was noted.

### Tissue temperature recording

For tissue temperature recording, type T thermocouple probes and/or fiber optic temperature probe were implanted into the adipose tissue of mice under anesthesia (Urethane at 1.3 g kg^−1^). Device implantation was performed according to the procedures described in previous studies^10,40^. Tissue temperature was recorded by TC-2000 Meter (Sable Systems International) or Reflex fiber signal conditioner (Neoptix). The Neoptix T1 fiber optic temperature probe has a response time of less than 500 ms and the size of the sensitive area is 300 microns in diameter. Temperature recording was initiated once tissue temperature was stabilized prior to light-pulses.

### Metabolic analyses

Whole-body energy expenditure and locomotor activity (beam break counts) were measured by using a comprehensive lab animal monitoring system (CLAMS) (Columbus Instruments). For the study of stimulation with optogenetic wireless implants, devices were implanted into the inguinal WAT of Adipo-*ChR2* mice and the littermate control mice before CLAMS analysis. Five days after the implantation, whole-body oxygen consumption rate (VO_2_), locomotor activity, and food intake were monitored at thermoneutrality (30 °C) by CLAMS. During the study, VO_2_ was measured before and after optogenetic stimulation at 10 Hz with 5-ms pulses for 10 min. For the study of β3-AR agonist (CL-316,243) administration, VO_2_ was measured before and after a single injection with CL-316,243 at 0.01 mg kg^−1^ or saline in wild-type mice at 14–16 weeks old at thermoneutrality (30 °C). The measurement was the average values of VO_2_ for 30 min before and after optogenetic stimulation or a i.p. injection of CL-316,243 or saline. Fat mass and lean mass were measured by Body Composition Analyzer EchoMRI (Echo Medical Systems). For glucose tolerance test experiments, after 6 h of fasting, the mice were injected intraperitoneally with glucose (2.0 g kg^−1^). To measure liver triglyceride contents, liver tissues were homogenized in 350 μl ethanolic KOH (100% EtOH: 30% KOH = 2:1) and incubated at 55 °C overnight. Subsequently, tissue lysates were brought volume to 1 ml with 50%EtOH. After centrifugation, the supernatant was incubated with 1 M MgCl_2_ on ice for 10 min. Amounts of triglycerides were determined by an Infinity Triglycerides kit (Thermo).

### Administration of AAV vectors

Adipo-*Serca2* KO mice (14–18 weeks old) and age-matched wild-type mice were treated with β3 adrenergic receptor agonist (CL-316, 243) at 1 mg kg^−1^ for 5 days. Subsequently, AAV expressing *ChR2* (AAV8-EF1a-hChR2 (H134R)-EYFP; the Neuroscience Gene Vector and Virus Core of Stanford University) or control GFP (AAV8-CAG-EGFP) were injected into the inguinal WAT of mice (viral titer: 8.5 × 10^9^ GC per mice). AAV was injected to six locations in the inguinal WAT to equally distribute the expression of ChR2 or GFP (5 μl volume at the viral tire of 1.7 × 10^9^ GC μl^−1^ per injection). Fourteen days after the AAV injection, tissue temperature was recorded in response to optogenetic light pulses. After temperature recording, the expression of ChR2 was confirmed by measuring the mRNA expression by qRT-PCR.

### Virus construction

Lentiviral vectors were obtained from Add-gene (pLenti-EF1a-hChR2(H134R)-EYFP-WPRE, Catalog #20942) and GeneCopoeia (shRNA targeting *Atp2a2*, CS-MSH028715-33-LVRU6GH; scrambled control, CSHCTR001-LVRU6GH). HEK293T cells were seeded at 70,000 cells per cm^2^ in 15 cm tissue culture dishes in 20 ml media (DMEM, 10% FBS) and incubated overnight at 37 °C and 5% CO_2_. Twenty-four hours after cell plating, 12 µg of lentiviral transfer vector was transfected together with 7 µg psPAX2 (Addgene #12260) and 3 µg pMD2.G (Addgene #12259) using 50 µl jetPRIME transfection reagent (Polyplus). Seventy-two hours after transfection, cell culture media were collected and filtered. Lentivirus purification was performed at UCSF Viral Core. Beige adipocytes were incubated in the virus overnight in the medium containing 6 μg ml^−1^ polybrene. Blasticidin at 10 µg ml^−1^ or hygromycin at 200 µg ml^−1^ was used for selection.

### Cell culture

*Ucp1* KO beige adipocytes were generated by immortalizing the stromal vascular fraction (SVF) from the inguinal WAT of *Prdm16* Tg x *Ucp1* KO mice^10^. SERCA2-depleted beige adipocytes were generated by infecting lentiviral shRNA targeting mouse *Atp2a2* (CS-MSH028715-33-LVRU6GH) to immortalized beige adipocytes in our previous study^10^. Beige adipocyte differentiation was induced by treating confluent preadipocytes with DMEM containing 10% FBS, 0.5 mM isobutylmethylxanthine, 125 nM indomethacin, 2 μg ml^−1^ dexamethasone, 850 nM insulin, and 1 nM T3, and 0.5 μM rosiglitazone^41^. Two days after induction, cells were switched to a maintenance medium containing 10% FBS, 850 nM insulin, 1 nM T3, and 0.5 μM rosiglitazone.

### Intracellular Ca^2+^ flux assays

Wild-type, *Ucp1* KO, and *Serca2*-depleted beige adipocytes expressing vector or *ChR2* were differentiated on collagen-coated glass-bottom dishes according to the beige fat differentiation protocol^41^. Intercellular Ca^2+^ levels were determined by using the Fluo-8 No Wash Calcium Assay kit (Abcam, ab112129). Differentiated adipocytes were incubated with Fluo-8 in calcium-free Hanks’ balanced salt solution (HHBS) at 37 °C for 30 min and subsequently incubated at room temperature for an additional 30 min. Subsequently, the buffer was replaced by HHBS, including 1 mM CaCl_2_, followed by the intracellular Ca^2+^ flux assays. For experiments using Ca^2+^ channel inhibitors, differentiated beige adipocytes expressing *ChR2* were incubated with L-type Ca^2+^ channel blocker (isradipine at 10 µM), R-type Ca^2+^ channel blocker (SNX-482 at 100 nM), or T-type Ca^2+^ channel blocker (NNC55-0396 at 3 µM), RyR2 receptor blocker (Ryanodine at 100 µM) or IP_3_R blocker (2-APB at 50 µM) for 90 min prior to optogenetics light stimulation. Intracellular Ca^2+^ level was monitored using a Revolve microscope (ECHO Laboratories) equipped with a 20× objective. The fluorescence was detected by the fluorescence filter Cube 2002 (ECHO Labolatories: Excitation 470/40 nm, dichroic 495 nm, emission 525/50 nm). Immediately after optogenetic light stimulation or adding noradrenaline at 1 µM, fluorescence intensity of differentiated adipocytes was monitored. Optogenetic light stimulation was delivered at 10 Hz with 5-ms width for 30 s. Image analyses were performed using the ImageJ software (https://imagej.nih.gov/ij/download.html). Specifically, the circular regions of interest (ROI) was placed onto individual differentiated adipocytes. Time-course changes in intracellular Ca^2+^ levels were recorded by quantifying the pixel intensity of the ROI and expressed as delta fluorescence ratio *F*/*F*0 or delta *F*/*F*0 relative to control, where *F* is the fluorescence intensity at a given time and *F*0 is the initial resting fluorescence intensity prior to stimuli. Background signal (signal from the area without cells) was subtracted from all data.

### Cellular oxygen consumption assays (OCR)

Oxygen consumption rate (OCR) in cultured adipocytes was measured using the Seahorse XFe Extracellular Flux Analyzer (Agilent) in a 24-well plate. For the measurement of NA-induced OCR, differentiated adipocytes were incubated in the XF assay media in the presence or absence of NA at 1 µM. For the measurement of optogenetics-induced cellular respiration, OCR in differentiated adipocytes expressing vector or *ChR2* was measured in the XF assay media before and after optogenetics stimulation. Cells were stimulated with optogenetics light at 10 Hz with 50-ms width for 10 min at 37 °C. To inhibit L-type Ca^2+^ channel, cells were incubated with isradipine at 10 µM or vehicle for 90 min prior to light stimulation. Light-induced OCR (%) values were calculated by OCR after light stimulation normalized by basal OCR prior to light stimulation. The XF assay medium was supplemented with 1 mM sodium pyruvate, 2 mM GlutaMax^TM^-I, and 25 mM glucose.

### Glucose oxidation

Differentiated adipocytes were incubated in DMEM containing 2% FBS for 2 h. After washing in PBS, cells were incubated in 1 ml of KRB/HEPES buffer that contained 1.8 mM CaCl_2_, 2% BSA, 15 mM glucose, 200 nM adenosine, and 0.5 μCi [1-^14^C] glucose. Cells received optogenetic light stimulation on a board with 24 LED’s at 10 Hz with 50-ms width for 30 min at 37 °C. Subsequently, 350 μl of 30% hydrogen peroxide was added in each well, and [^14^C] CO_2_ from each well was trapped in the smears supplemented with 300 μl of 1 M benzethonium hydroxide solution at room temperature for 20 min. Glucose oxidation was quantified by counting the radioactivity of trapped [^14^C] CO_2_ using a scintillation counter.

### Histology

For hematoxylin and eosin (H&E) staining, adipose tissues were fixed in 4% paraformaldehyde, followed by 70% ethanol. Tissues were embedded in paraffin, sectioned at 5 µm, and stained with H&E. Adipocyte images were acquired using a Revolve microscope (ECHO Laboratories), and the cellular size was quantified by using the Image J software. For the adipocyte size quantification, we measured at least 500 cells per mouse.

### Quantitative RT-PCR

Total RNA was extracted from the adipose tissues using RiboZol reagents (AMRESCO) followed by the RNeasy mini-kit (Qiagen Inc., Valencia, CA). cDNA was synthesized by iScript cDNA Synthesis kit (BioRad) according to the provided protocol. Quantitative real-time PCR (qRT-PCR) was performed using an ABI ViiA™7 PCR cycler (Applied Biosystems). Each sample was run in duplicate, and the expression levels of each gene were normalized to *36B4*. Relative mRNA levels were determined by the ΔΔ Ct method and normalized to an internal calibrator specific to each gene using the formula 2^−ΔΔCT^. Primer sequences are provided in Supplementary Table 1.

### Statistical analysis

Statistical analyses were performed using GraphPad Prism 7.0 (GraphPad Software, Inc., La Jolla, CA). All data are expressed as means ± SEM. Comparisons between the two groups were analyzed using the paired or unpaired *t-*test as appropriate. One-way or two-way ANOVA, followed by Tukey’s test was used for multiple group comparisons. Two-way repeated-measures ANOVA followed by post-hoc paired/unpaired *t*-test with Bonferroni’s correction was used for the comparisons of repeated measurements. *P* values below 0.05 were considered significant throughout the study.

## Supplementary information

Supplementary Information

Description of Additional Supplementary Information

Supplementary Movie 1

Supplementary Movie 2

## Data Availability

The source data underlying Figs. 1d–m, 2b–d, f, g, 3a, b, 4a–d, f, and Supplementary Figs. 1a, b, d–g, 2a, b, d–f, 3a–d, and 4a–g are provided as a Source Data file. All other relevant data of this study are available from the corresponding authors upon reasonable request. A reporting summary is available as a Supplementary Information file.

## Source data

Source data
