# Supplementary material for: Wireless optogenetics protects against obesity via stimulation of non-canonical fat thermogenesis
Source: Nat Commun. 2020 Apr 7;11:1730. doi: 10.1038/s41467-020-15589-y (PMC7138828; doi:10.1038/s41467-020-15589-y)
Supplement: Supplementary file 1 — Supplementary Information [file 41467_2020_15589_MOESM1_ESM.pdf]

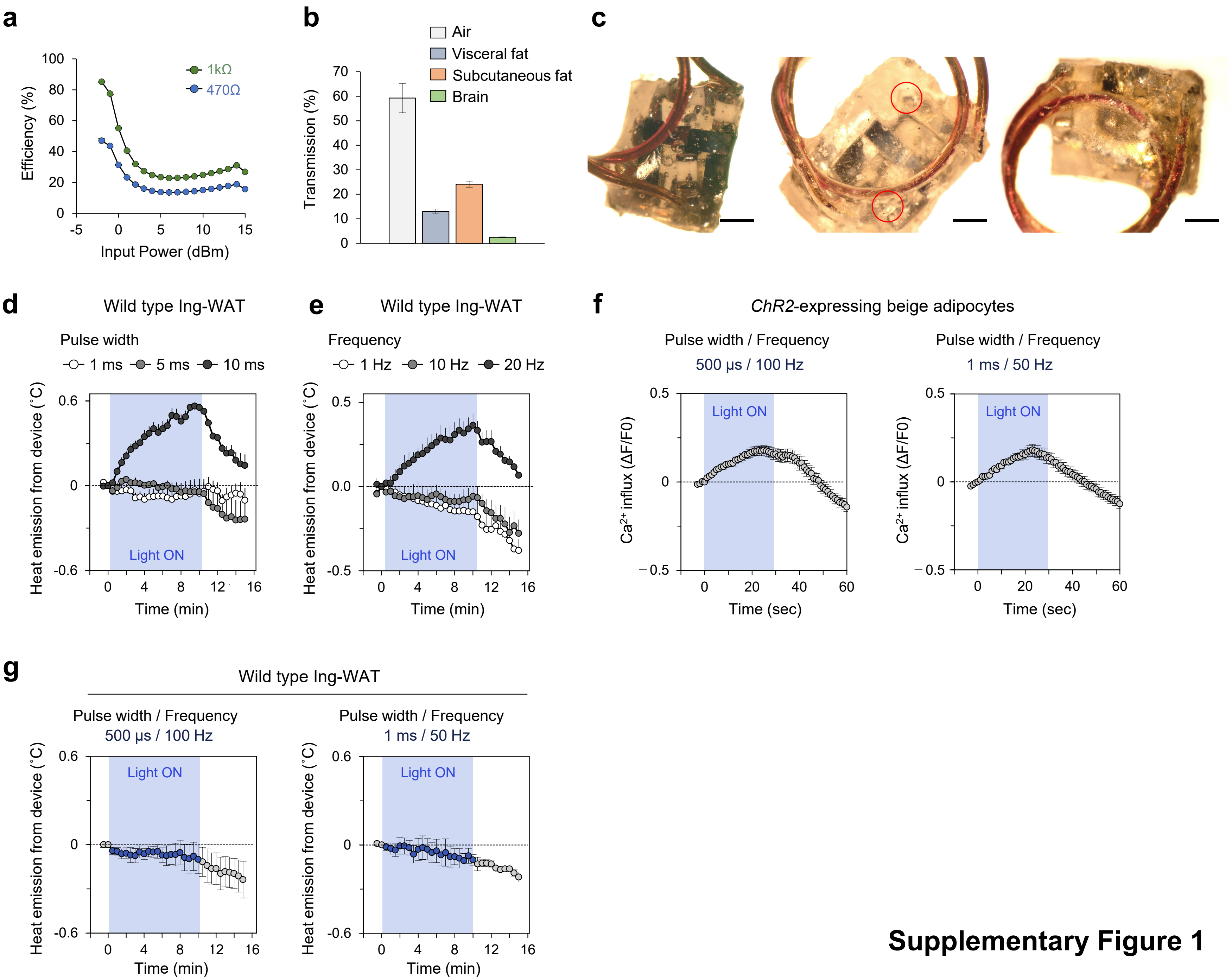

Supplementary Figure 1

Supplementary Figure 1. Optimization of light stimulation by the wireless optogenetic implant.

a. Rectifier efficiency versus input power for two loading conditions. n=3. **b**. Optical properties of the adipose tissues and brain. Based on this data, the attenuation coefficients were calculated (Adipose tissue, μ'_s=0.8 mm^{-1}; Brain, μ'_s=1.6 mm^{-1}). n=3. **c**. Demonstrates the degradation of the encapsulated implant in 20 mM Hydrogen Peroxide at 60 ˚C. Epoxy coating before submerging in hydrogen peroxide solution (left panel). Epoxy coating without Parylene C after 5 days in solution. Red circles represent some intrusion of liquid (middle panel). No intrusion of liquid under epoxy coating with Parylene C after 5 days in solution. (right panel). **d**. Heat emission from the device following optogenetic light stimulations with indicated pulse width. n=5. **e**. Heat emission from the device following optogenetic light stimulations with indicated pulse frequency. Mice stimulated with 10-Hz frequency, n=3; with 1-Hz or 20-Hz frequencies, n=4. **f**. Real-time intracellular Ca^{2+} influx changes following optogenetic light stimulation with indicated pulse width and frequency. Cells stimulated with 500-µs pulse width and 100-Hz frequency, n=90; with 1-ms pulse width and 50-Hz frequency, n=105. **g**. Heat emission from device following optogenetics light stimulation with indicated pulse width and frequency. Mice stimulated with 500-µs pulse width and 100-Hz frequency, n=4; with 1-ms pulse width and 50-Hz frequency, n=3. All Data are expressed as means ± s.e.m.

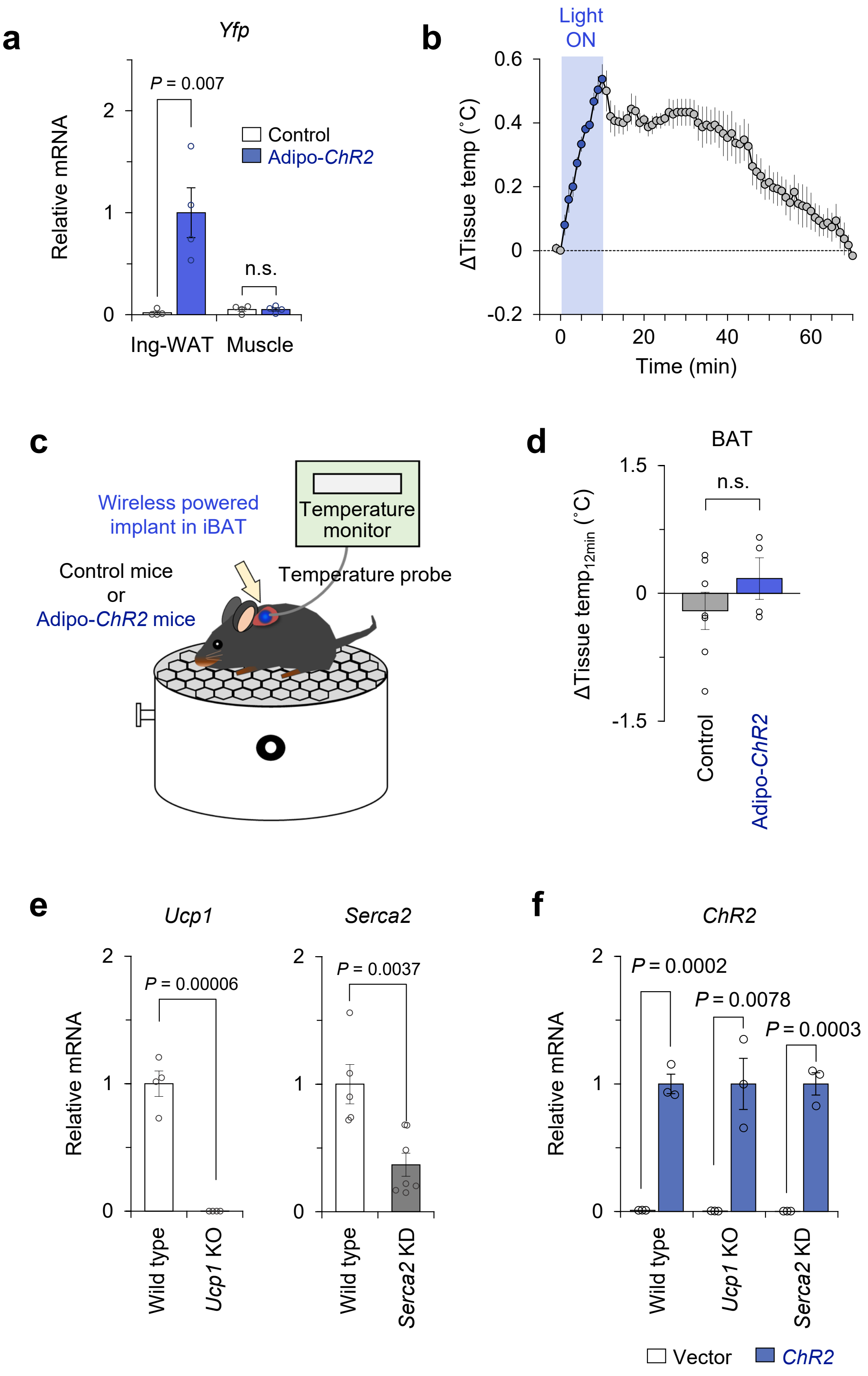

Supplementary Figure 2

Supplementary Figure 2. Characterization of Adipo-*ChR2* mice.

a. mRNA expression of *Yfp* in the inguinal WAT and skeletal muscle of Adipo-*ChR2* and littermate controls. mRNA expression relative to *36B4*. n=4. **b**. Real-time changes in the inguinal WAT temperature of Adipo-*ChR2* mice following optogenetics light stimulation. n=5. **c**. Schematic illustration of the iBAT temperature recording following optogenetic light stimulation. **d**. Quantification of light-stimulated iBAT thermogenesis of Adipo-*ChR2* and littermate controls in (c). Control, n=7; Adipo-*ChR2*, n=4. **e.** mRNA expression of *Ucp1* (left) and *Serca2* (right) in the indicated beige adipocytes. mRNA expression relative to *36B4*. *Ucp1*; Wild-type, n=4; *Ucp1* KO, n=4; *Serca2*; Wild-type, n=5; *Serca2*KD, n=7. **f**. mRNA expression of *ChR2* in indicated beige adipocytes. mRNA expression relative to *36B4*. n=3. Data were analyzed by unpaired two-sided *t*-test (a, d, and e-f). All Data are expressed as means ± s.e.m. n.s., not significant.

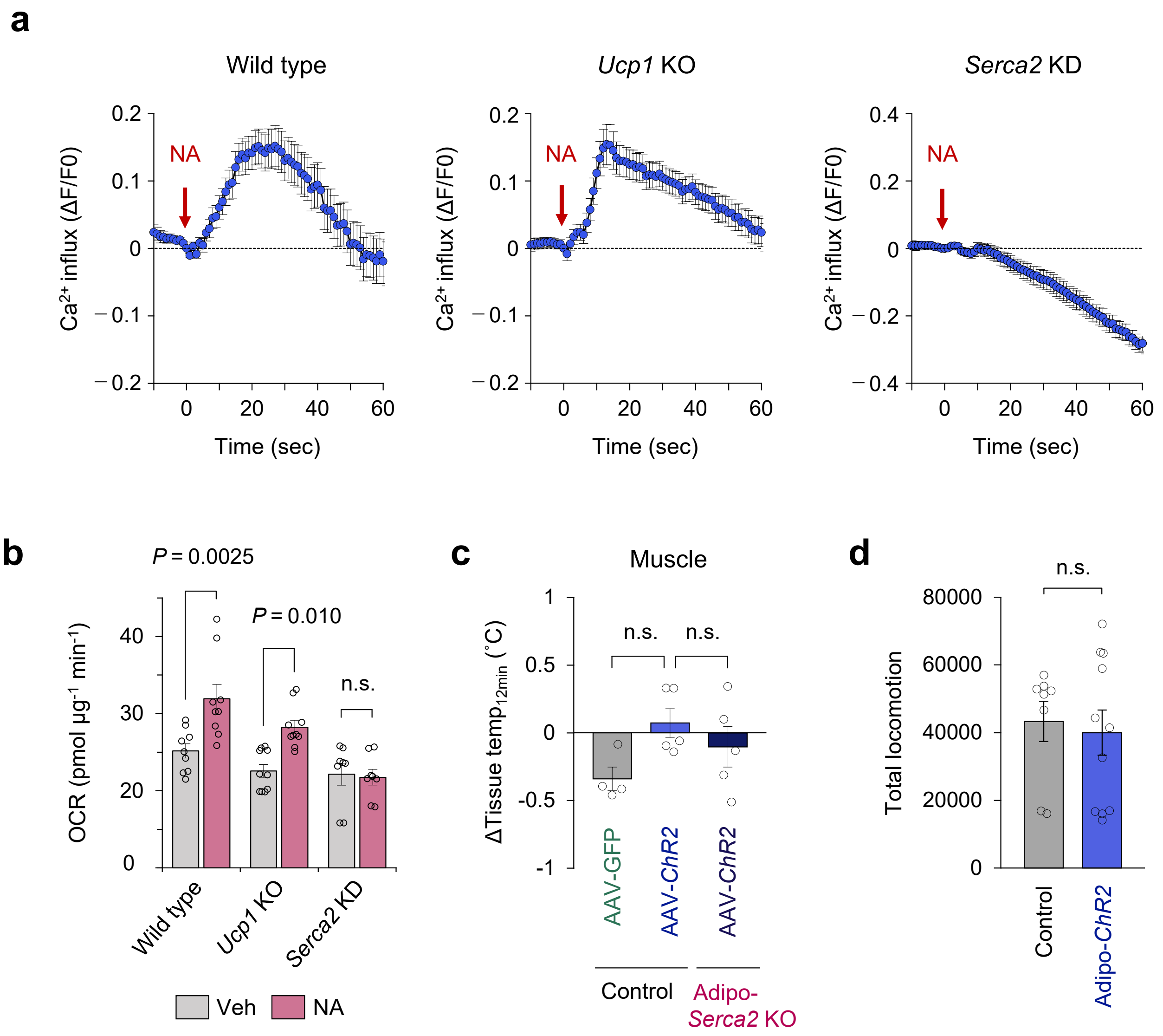

Supplementary Figure 3

Supplementary Figure 3. The requirement of SERCA2 for adipose tissue thermogenesis.
a. Real-time changes in intracellular Ca^{2+} influx in wild-type (left), *Ucp1* KO (middle), and *Serca2* depleted beige adipocytes (right) in response to noradrenaline (NA) treatment (shown by red arrows). Wild-type, n=85; *Ucp1* KO, n=70; *Serca2*KD, n=85. **b**. Oxygen consumption rate (OCR) in wild-type, *Ucp1* KO, and *Serca2* depleted beige adipocytes. Wild-type, n=9 for both groups; *Ucp1* KO, n=10 for both groups; *Serca2*KD, n=8 for both groups. **c.** Quantification of light-stimulated thermogenesis in the skeletal muscle of the indicated mice. Control with AAV-GFP, n=4; Control with AAV-*ChR2*, n=5; Adipo- *Serca2* KO with AAV-*ChR2*, n=5. **d**. Locomotor activity of Adipo-*ChR2* mice and littermate controls. All mice were stimulated with optogenetics wireless implant at 10 Hz with a 5-ms pulse width for 10 min per day. Control, n=8; Adipo-*ChR2*, n=11. Data were analyzed by two-way ANOVA (b) or one-way ANOVA (c) by Tukey's post hoc test, or unpaired two-sided *t*-test (d). All Data are expressed as means ± s.e.m. n.s., not significant.

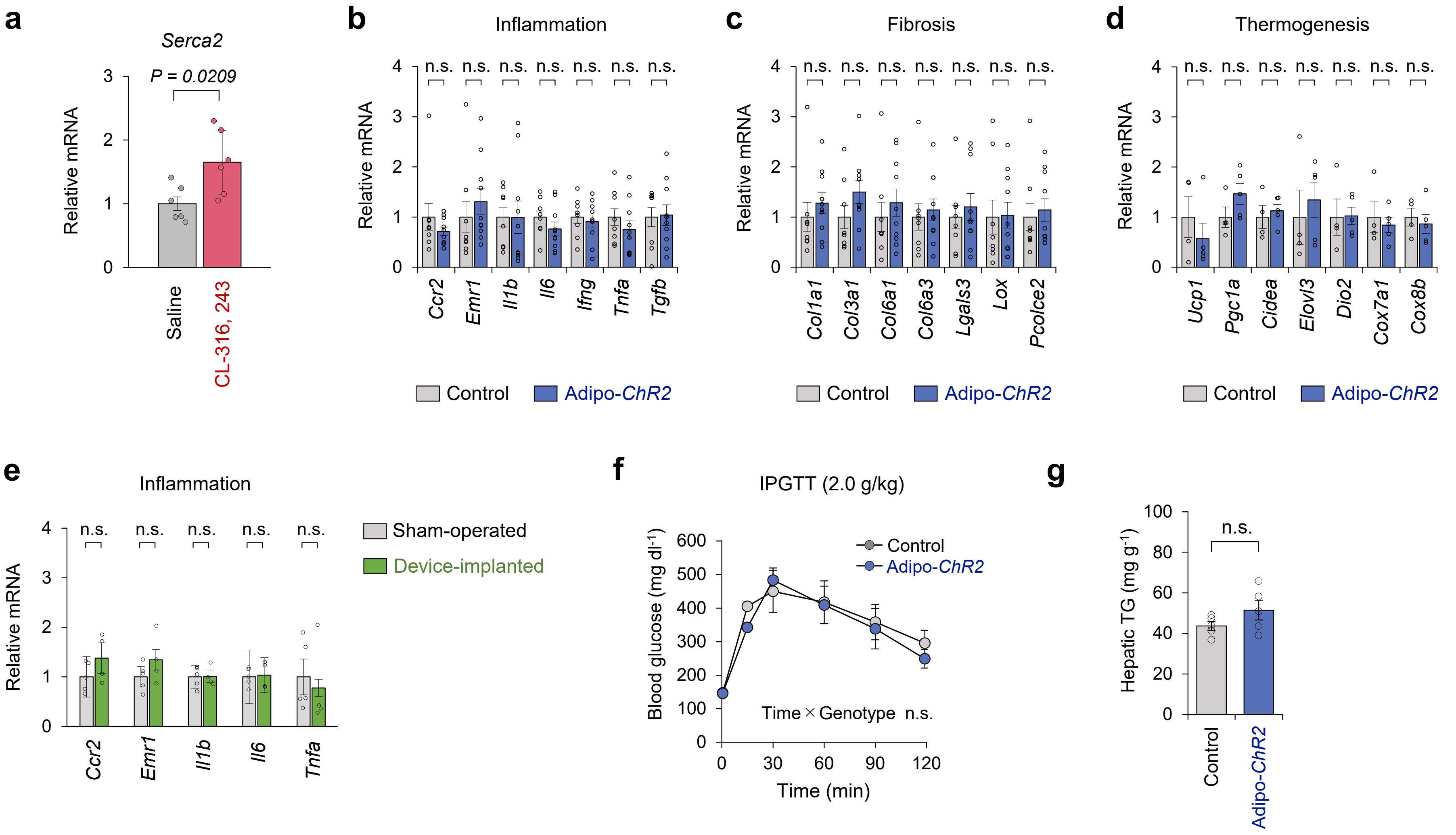

Supplementary Figure 4

Supplementary Figure 4. The effect of optogenetic stimulation on adipose tissues.
a. mRNA expression of *Serca2* in the inguinal WAT of wild-type mice following CL-316, 243 treatment. The mice at 10 weeks old were treated with saline or CL-316, 243 at 1 mg kg^{-1} for 5 days. mRNA expression relative to *36B4*. n=6. **b-d.** mRNA expression of pro-inflammatory genes (b), pro-fibrosis genes (c), and thermogenesis genes (d) in the inguinal WAT of Adipo-*ChR2* mice and littermate controls. mRNA expression was relative to *36B4*. Control, n=8 for inflammation genes and fibrosis genes, n=4 for thermogenesis genes; Adipo-*ChR2*, n=10 for inflammation genes and fibrosis genes, n=5 for thermogenesis genes. **e**. mRNA expression of pro-inflammatory genes in the inguinal WAT of mice that received wireless optogenetic devices or sham-operated mice. Fourteen days after sham operation or implanted operation, the inguinal WAT was harvested. Sham-operated, n=5; Device-implanted, n=4. mRNA expression was relative to *36B4*. **f**. Glucose tolerance test in Adipo-*ChR2* mice and littermate controls. Mice received optogenetic light stimulation at 10 Hz with a 5-ms pulse width for 10 min for 19 days on a HFD. n=5. **g**. Liver triglyceride contents in Adipo-*ChR2* mice and littermate controls. n=5. Data were analyzed by unpaired two-sided *t*-test (a-e, and g) or two-way repeated-measures ANOVA (f). All Data are expressed as means ± s.e.m. n.s., not significant.

Supplementary Table 1. Primer sequences used for quantitative RT-PCR.

Gene	Forward	Reverse
Ccr2	ATCCACGGCATACTATCAACATC	TCGTAGTCATACGGTGTGGTG
ChR2	CAATGTTACTGTGCCGGATG	ATTTCAATGGCGCACACATA
Cidea	ATCACAACTGGCCTGGTTACG	TACTACCCGGTGTCCATTTCT
Col1a1	GCTCCTCTTAGGGGCCACT	CCACGTCTCACCATTGGGG
Col3a1	CTGTAACATGGAAACTGGGGAAA	CCATAGCTGAACTGAAAACCACC
Col6a1	CTGCTGCTACAAGCCTGCT	CCCCATAAGGTTTCAGCCTCA
Col6a3	GCTGCGGAATCACTTTGTGC	CACCTTGACACCTTTCTGGGT
Cox7a1	CAGCGTCATGGTCAGTCTGT	AGAAAACCGTGTGGCAGAGA
Cox8b	GAACCATGAAGCCAACGACT	GCGAAGTTCACAGTGGTTCC
Dio2	CAGTGTGGTGCACGTCTCCAATC	TGAACCAAAGTTGACCACCAG
Elovl3	TCCGCGTTCTCATGTAGGTCT	GGACCTGATGCAACCCTATGA
Emr1	CTTGGCTATGGGCTTCCAGTC	GCAAGGAGGACAGAGTTTATCGTG
Ifng	ACAGCAAGGCGAAAAAGGATG	TGGTGGACCACTCGGATGA
Il1b	ATGCCACCTTTTGACAGTGAT	AGCCCTTCATCTTTTGGGGT
Il6	CCCCAATTTCCAATGCTCTCC	GGATGGTCTTGGTCCTTAGCC
Lgals3	TGCTGGTTCCAGGGACTCAA	CCACCGGCCTCTGTAGAAGA
Lox	CAGCCACATAGATCGCATGGT	GCCGTATCCAGGTCGGTTC
Pcolce2	TGTGGCGGCATTCTTACCG	CCCTCAGGAACTGTGATTTTCCA
Pgc1a	AGCCGTGACCACTGACAACGAG	GCTGCATGGTTCTGAGTGCTAAG
Serca2	TCGACAGGACAGAAAGAGTGTG	AAACTGAATTCAACTCACCAGC
Tgfb	CTCCCGTGGCTTCTAGTGC	GCCTTAGTTTGGACAGGATCTG
Tnfa	ATGGCCTCCCTCTCATCAGT	TTTGCTACGACGTGGGCTAC
Ucp1	CACCTTCCCGCTGGACACT	CCCTAGGACACCTTTATACCTAATGG
Yfp	AGCTGAAGGGCATCGACTTC	AGCAGGACCATGTGATCGC
36B4	TCCAGGCTTTGGGCATCA	CTTTATCAGCTGCACATCACTCAGA
