# Supplementary material for: Wireless optogenetics protects against obesity via stimulation of non-canonical fat thermogenesis
Source: Nat Commun. 2020 Apr 7;11:1730. doi: 10.1038/s41467-020-15589-y (PMC7138828; doi:10.1038/s41467-020-15589-y)
Supplement: Supplementary file 2 — Description of Additional Supplementary Information [file 41467_2020_15589_MOESM2_ESM.docx]

**Description of Additional Supplementary Files**

**File name: Supplementary Movie 1**

**Description:** A freely behaving mouse implanted with the wireless optogenetics device. A wireless optogenetics device was implanted in the inguinal WAT of Adipo-ChR2 mice. Optogenetics stimulation was delivered at 10-Hz frequency with 5-ms pulse width. The mirror image shows the optogenetics device that was implanted into the left side of inguinal WAT.

**File name: Supplementary Movie 2**

**Description:** Ca2+ imaging in beige adipocytes expressing ChR2 following optogenetics light stimulation. Differentiated beige adipocytes expressing ChR2 were stimulated by optogenetics blue light at 10 Hz with 5-ms pulse width. The movie is played back at double speed (2x). At the end of the video, a representative image of differentiated adipocytes before and after optogenetic light stimulation was shown.
